# Combined intervention of *Akkermansia muciniphila* and sodium butyrate ameliorates oxaliplatin-induced peripheral neuropathy by suppressing neuroinflammation and reducing serum neurofilament light chain

**DOI:** 10.3389/fimmu.2026.1787012

**Published:** 2026-03-04

**Authors:** Dan Xu, Danting Fu, Jian Chen, Qiang Hu, Yanning Wang, Jinfeng Wu

**Affiliations:** 1 The Integrated Traditional Chinese and Western Medicine School of Clinical Medicine, Zhejiang Chinese Medical University, Hangzhou, China; 2 Department of Gastrointestinal Surgery, Tongde Hospital of Zhejiang Province Affiliated to Zhejiang Chinese Medical University, Hangzhou, China; 3 Department of Experimental Animals, Zhejiang Academy of Traditional Chinese Medicine, Hangzhou, China

**Keywords:** *Akkermansia muciniphila*, chemotherapy-related neuropathic pain, neurofilament light chain, oxaliplatin-induced peripheral neuropathy, sodium butyrate

## Abstract

**Introduction:**

Oxaliplatin-induced peripheral neuropathy (OIPN) is a major dose-limiting neurotoxic side effect that severely disrupts quality of life and compromises antitumor efficacy. Despite its clinical prevalence, effective neuroprotective strategies remain elusive, and diagnosis largely relies on subjective symptom reporting rather than objective pathological assessment. Emerging evidence suggests that targeting the gut-peripheral nerve axis offers a novel therapeutic avenue/approach. Therefore, this study primarily aimed to investigate the combined neuroprotective efficacy of *Akkermansia muciniphila* (*A. muciniphila*) and sodium butyrate in a rat model of OIPN. Furthermore, we evaluated the potential clinical utility of serum neurofilament light chain (NfL) for the early diagnosis, severity assessment, and therapeutic monitoring of OIPN.

**Methods:**

An OIPN rat model was established via tail vein injection of oxaliplatin. Sprague-Dawley rats were randomized by body weight into five groups: control, model, *A. muciniphila* treatment, sodium butyrate (NaB) treatment, and combined *A. muciniphila* + NaB treatment. Outcome measures encompassed longitudinal body weight monitoring, behavioral assessments (mechanical allodynia and cold allodynia), histopathological examination of the lumbar dorsal root ganglia (DRG) and intraepidermal nerve fiber density (IENFD) in hind paws, as well as quantification of serum inflammatory cytokines and NfL levels.

**Results:**

Oxaliplatin administration induced a progressive neuropathy characterized by significant weight loss, mechanical allodynia, and cold allodynia. Histopathologically, neuronal atrophy, axonal degeneration, demyelination, and inflammatory infiltration were observed in the DRG and peripheral nerves, accompanied by a marked reduction in IENFD. Serological analysis indicated a systemic pro-inflammatory shift (elevated IL-6, IL-1β, and TNF-α; decreased IL-10) alongside a substantial elevation in NfL, a specific biomarker of axonal injury. All therapeutic interventions attenuated neuropathic pain, ameliorated structural nerve damage, modulated inflammatory cytokine profiles, and reduced serum NfL levels. Notably, combination therapy (*A. muciniphila* + NaB) exerted the most pronounced neuroprotective effects, particularly regarding late-phase pain control, nerve fiber preservation, and the normalization of NfL levels.

**Conclusions:**

Our findings confirm that oxaliplatin triggers complex pathological alterations, including pain hypersensitivity, neuroinflammation, and axonal injury. The combinatorial treatment with *A. muciniphila* and sodium butyrate exerted potent neuroprotective effects, significantly relieving mechanical and cold allodynia while suppressing systemic inflammation. Intriguingly, these therapeutic benefits were closely mirrored by a marked reduction in serum NfL levels. Collectively, our data not only validate the efficacy of this gut-microbiota-targeted strategy but also establish serum NfL as a sensitive, objective metric for monitoring OIPN severity and therapeutic response.

## 1 Introduction

According to GLOBOCAN 2022 statistics, approximately 20 million new cancer cases were diagnosed worldwide in 2022, with projections estimating a rise to 35 million by 2050 (1). Colorectal cancer ranks as the third most prevalent malignancy and the second leading cause of cancer-related mortality, representing a substantial public health challenge (2).

Currently, oxaliplatin-based chemotherapy regimens constitute the standard first-line treatment for most gastrointestinal malignancies, particularly colorectal and gastric cancers, significantly improving patient survival outcomes (3). However, its clinical utility is frequently constrained by a major dose-limiting toxicity: oxaliplatin-induced peripheral neuropathy (OIPN). OIPN is characterized by progressive peripheral nerve damage, resulting in sensory and motor dysfunction. Studies indicate that approximately 85%–95% of patients develop acute OIPN during treatment, with over half progressing to chronic neuropathy (4). Notably, symptoms in some patients continue to exacerbate for months following treatment cessation (the coasting phenomenon), potentially leading to long-term, irreversible neurological deficits (5). OIPN not only induces pain, paresthesia, and functional impairment—severely compromising quality of life—but also frequently necessitates dose reduction or treatment discontinuation, thereby undermining antitumor efficacy (6–8).

**Figure 1:**
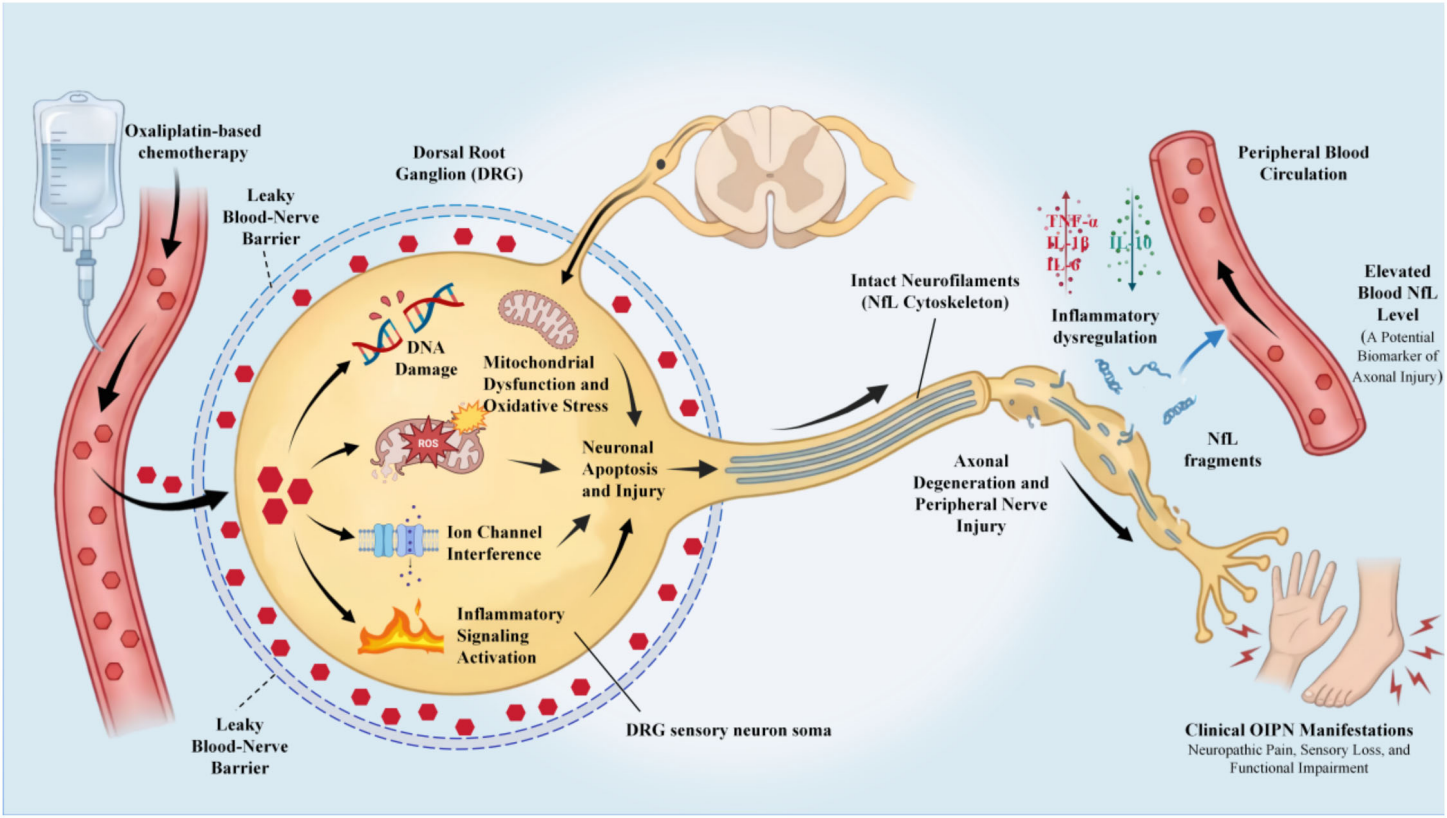
Mechanism of oxaliplatin-induced peripheral neuropathy (OIPN) and NfL release. This is a conceptual schematic (not patient data or experimental imaging). The figure was created with BioRender.com and assembled in Microsoft PowerPoint. The authors take full responsibility for the integrity and accuracy of the figure and its content.

The dorsal root ganglion (DRG) represents the primary target of oxaliplatin-induced neurotoxicity. Due to the high permeability regarding the blood-nerve barrier, the DRG is highly susceptible to drug accumulation within sensory neuron cell bodies (9, 10). Oxaliplatin triggers a cascade of pathological events, including DNA damage, ion channel dysfunction, oxidative stress, mitochondrial impairment, and the activation of inflammatory signaling pathways (**Figure 1**). These processes ultimately culminate in DRG neuronal apoptosis, axonal degeneration, and peripheral nerve fiber injury, clinically manifesting as neurological dysfunction and neuropathic pain (11, 12).

Currently, OIPN diagnosis relies predominantly on subjective symptom reporting and clinical behavioral scales. However, these methods are susceptible to inter-individual variability, exhibit limited reproducibility, and fail to objectively quantify axonal damage at early stages (13). Consequently, there is an urgent clinical need for blood-based biomarkers capable of providing an early, objective, and quantitative assessment of neural axonal injury, thereby facilitating OIPN prediction, diagnosis, and therapeutic monitoring (7).

In recent years, neurofilament light chain (NfL) has garnered distinct attention as a promising blood-based biomarker for axonal injury (14). As a critical structural component of the neuronal cytoskeleton, NfL is released into the extracellular space upon axonal damage, subsequently entering the cerebrospinal fluid and peripheral circulation (4). Peripheral blood NfL levels correlate closely with the magnitude of central and peripheral nervous system injury and have been validated for diagnostic and prognostic assessment in various neurological disorders, including Alzheimer’s disease, multiple sclerosis, and stroke (15–19). However, current research on NfL in chemotherapy-induced peripheral neuropathy has been largely confined to static correlational analyses, and its clinical utility in the dynamic monitoring of treatment response remains to be elucidated.

Emerging evidence implicates the gut-brain axis as a critical therapeutic target for OIPN, as chemotherapy disrupts gut microbiota homeostasis, leading to systemic inflammation that exacerbates neuropathic pain (20, 21). Therefore, strategic modulation of this axis presents a promising avenue for intervention. In this study, *Akkermansia muciniphila* (*A. muciniphila*) and sodium butyrate (NaB) were selected as representative therapeutic interventions targeting distinct but complementary aspects of the gut-immune-nerve axis (21). *A. muciniphila*, a keystone commensal bacterium, was chosen to reconstruct the compromised gut microbiota, enhance intestinal barrier function, and inhibit the systemic translocation of pro-inflammatory lipopolysaccharides (LPS) (22–24). Furthermore, *A. muciniphila* promotes the production of downstream metabolites, such as butyrate, establishing a metabolic cross-feeding network that reinforces the gut-brain defense against chemotherapy-induced neurotoxicity (25). NaB was selected as a chemical intervention representing the downstream functional metabolites of the gut flora. As a histone deacetylase inhibitor, NaB was included to evaluate its direct neuroprotective properties, specifically the suppression of spinal microglial activation and pro-inflammatory cytokine release, thereby attenuating mechanical allodynia (25–28), as well as its potential to modulate nociceptive pathways via PPAR-α activation and CB1/opioid receptor signaling (29).

Based on this premise, the present study aims to elucidate the therapeutic potential of a combined intervention with *A. muciniphila* and sodium butyrate in alleviating OIPN. Specifically, we focus on the mechanisms involving the “gut-immune-nerve axis,” hypothesizing that this combination can suppress neuroinflammation, thereby attenuating neuropathic pain and ameliorating peripheral nerve injury. Furthermore, we investigated the utility of serum NfL as a pharmacodynamic biomarker to monitor the progression of neuronal damage and evaluate the efficacy of this gut-targeted neuroprotective strategy.

## 2 Materials and methods

### 2.1 Drugs and reagents

Oxaliplatin injection (50 mg/10 ml) was obtained from Sichuan Huiyu Pharmaceutical Co., Ltd. (Sichuan, China). *A. muciniphila* (ATCC^®^ BAA-835™; Product No. BNCC 323275) was supplied by BNCC^®^ (Henan Engineering Research Center of Industrial Microbiology, Henan, China). The bacterial suspension was stored at 4°C at a concentration of 2.0 × 10^9^ colony-forming units (CFU)/ml. NaB (CAS: 156-54-7; purity ≥ 98%) was sourced from Shanghai Macklin Biochemical Technology Co., Ltd. (Shanghai, China). Enzyme-linked immunosorbent assay (ELISA) kits targeting tumor necrosis factor-α (TNF-α; JX-1721A1), interleukin-1β (IL-1β; JX-1588A1), interleukin-6 (IL-6; JX-1731A1), interleukin-10 (IL-10; JX-1736A1), and neurofilament light chain (NfL; JX-11808A) were purchased from Yancheng Junxing Biotechnology Co., Ltd. (Yancheng, China).

### 2.2 Animals and grouping

This study was approved by the Laboratory Animal Welfare and Ethics Committee of the Zhejiang Academy of Traditional Chinese Medicine (Approval No. 2025002) and was conducted in strict compliance with the guidelines of the International Association for the Study of Pain (IASP).

**Figure 2:**
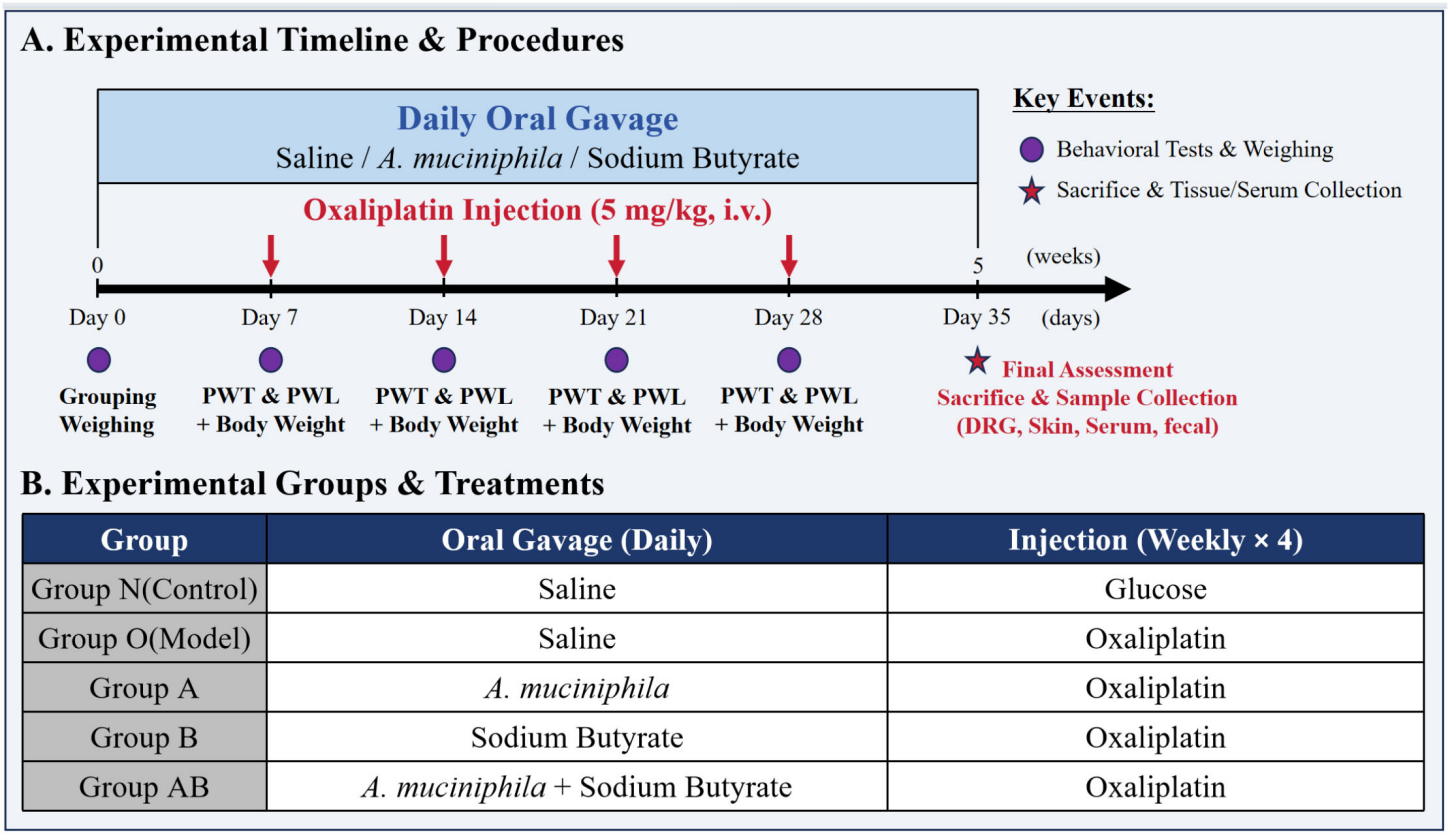
Intervention scheme and experimental flow chart in rats. **(A)** Experimental Timeline & Procedures. **(B)** Experimental Groups & Treatments (N, Normal control; O, Oxaliplatin model; A, Oxaliplatin + *A. muciniphila*; B, Oxaliplatin + Sodium Butyrate; AB, Oxaliplatin + *A. muciniphila* + Sodium Butyrate).

Twenty-five male Sprague-Dawley rats (6–8 weeks old, weighing 250–300 g) were obtained from Hangzhou Medical College, Zhejiang, China (Certificate No. 20250421Aazz01000180033). The animals were housed under specific pathogen-free (SPF) conditions at a controlled temperature of 22 ± 2°C and a relative humidity of 50 ± 10%, under a standard 12-hour light/dark cycle with ad libitum access to food and water. Following a one-week acclimatization period, the rats were weighed and randomly allocated into five experimental groups (n = 5 per group): normal control (Group N), OIPN model (Group O), *Akkermansia muciniphila* intervention (Group A), sodium Butyrate intervention (Group B), and combined *Akkermansia muciniphila* and sodium Butyrate intervention (Group AB), as illustrated in **Figure 2**.

### 2.3 Establishment of the OIPN rat model

While intraperitoneal injection has been the predominant route for oxaliplatin administration in previous studies, intravenous delivery more accurately replicates the clinical administration pathway, despite greater technical demands. Accordingly, this study employed tail vein injection (30). Oxaliplatin was dissolved in 5% glucose to a concentration of 2 mg/mL and administered intravenously at 5 mg/kg (0.25 mL/100 g) once weekly for four consecutive weeks. Group N received an isovolumetric injection of 5% glucose solution via the same route.

### 2.4 Treatment interventions

As detailed in the Introduction, *A. muciniphila* and sodium butyrate were selected to target the gut-immune-nerve axis. By using these agents, we aimed to confirm whether targeting the gut-immune-nerve axis could effectively mitigate oxaliplatin-induced neurotoxicity. For drug administration, the *A. muciniphila* suspension was stored at 4°C, thoroughly mixed, and diluted with phosphate-buffered saline (PBS). Rats received a daily oral gavage of 1×10^9^ CFU suspended in 0.5 mL PBS. NaB was dissolved in PBS and administered daily via oral gavage at a dose of 100 mg/kg (in a volume of 0.5 mL/rat). Rats in the treatment groups (Groups A, B, and AB) received the corresponding interventions via oral gavage once daily for 34 consecutive days (from Day 1 to Day 34). On Day 35, 24 hours after the final administration, all rats were sacrificed for sample collection.

### 2.5 Body weight and behavioral assessment

Body weight was monitored weekly from week 0 to week 5. Behavioral assessments for mechanical and cold allodynia were conducted on the first day of each week (weeks 1–5).

Rats were placed in individual transparent perspex boxes on an elevated wire mesh grid and allowed to acclimate for 30 minutes. A dynamic plantar aesthesiometer (Model KW-CT-1; Nanjing Calvin Biotechnology Co., Ltd., China) equipped with a 0.6 mm steel filament was used. The filament was applied vertically to the plantar surface of the left hind paw with increasing force. The force (in grams) at which the rat withdrew its paw was recorded as the Paw Withdrawal Threshold (PWT). Three measurements were taken for each rat with an inter-trial interval of 5 minutes, and the average was calculated.

Cold sensitivity was assessed using a cold plate apparatus (Model ZH-6C; Anhui Zhenghua Biological Instrument Co., Ltd., China) maintained at 4°C. Rats were placed individually on the cold surface, and the time elapsed until the first sign of nociceptive behavior (lifting, licking, or shaking of the hind paw) was recorded as the Paw Withdrawal Latency (PWL). To prevent tissue damage, a cut-off time of 30 seconds was imposed. If no response was observed within this limit, the latency was recorded as 30 seconds. A minimum interval of 10 minutes was allowed between tests to avoid sensitization.

### 2.6 Sample collection and processing

On day 35 of the experiment, rats were administered an overdose of Zoletil^®^50 via intraperitoneal injection to ensure deep anesthesia, followed by rapid cardiac blood collection after thoracotomy. Collected tissues included central plantar skin from the hind paws, DRG from the L4–L5 spinal segments. Blood samples were centrifuged to isolate serum, which was stored at –80°C until analysis.

### 2.7 Serum biomarker assays

Serum samples were collected at the end of the experiment (Day 35). The concentrations of TNF-α, IL-1β, IL-6, IL-10, and NfL were measured using rat-specific two-step sandwich ELISA kits according to the manufacturer’s instructions. All samples were analyzed in triplicate. Optical density (OD) was determined at 450 nm using a microplate reader (Infinite^®^ F50; Tecan Trading AG, Switzerland). Analyte concentrations were calculated based on their respective standard curves. The mean of the three technical replicates for each sample was used for statistical analysis.

### 2.8 Histopathological analysis

L4–L5 DRG tissues were harvested, fixed in 4% paraformaldehyde, and subjected to standard processing, including dehydration, paraffin embedding, and sectioning. Sections were stained with hematoxylin and eosin (H&E) and digitized using a panoramic tissue scanner (KF-PRO-120; KFBIO, China). Pathological alterations in DRG neurons—including soma shrinkage, nuclear pyknosis, interstitial edema, and inflammatory cell infiltration—were evaluated under light microscopy. Using Visiopharm software (Visiopharm A/S, Denmark), the number of inflammatory cells in each rat DRG sample was analyzed across three different sections or three non-overlapping fields of view (400× magnification). The analysis specifically quantified lymphocytes, macrophages, and neutrophils within the loose connective tissue (interstitium) distributed between neurons. The analysis workflow followed a standardized protocol involving region definition, phenotyping, and algorithmic quantification. First, Regions of Interest (ROIs) were automatically delineated along the tissue boundaries, with manual adjustments applied where necessary to ensure anatomical precision. Second, for signal detection, the AI algorithm analyzed fluorescent expressions on cell nuclei to establish positivity thresholds; these settings were saved and consistently applied across all sections within the same batch to ensure reproducibility. Third, cellular identification was achieved by detecting DAPI-stained nuclei and algorithmically expanding the boundaries to define the cytoplasmic area. Finally, the software computed quantitative parameters—including positive cell counts—via high-resolution stepwise calculation within the defined ROIs. To ensure data accuracy, a senior pathologist, blinded to the experimental grouping, performed a systematic re-evaluation of the H&E-stained DRG sections to validate the automated counts. In instances where the automated quantification deviated from the pathologist’s assessment, the manual counts were recorded as the final data.

### 2.9 Intraepidermal nerve fiber density

As the diagnostic gold standard for small fiber neuropathy recommended by the European Federation of Neurological Societies (31), IENFD was quantified. Plantar skin tissues were fixed, sectioned, and subjected to immunofluorescence staining using an antibody against the pan-neuronal marker PGP9.5 (Proteintech^®^). Images were acquired using a Pannoramic SCAN scanner (3D Histech, Hungary). Using Visiopharm software (Visiopharm A/S, Denmark), 3 non-overlapping fields of view (400× magnification) were selected for each rat to count the nerve fibers crossing the basement membrane, and the mean value was calculated.

### 2.10 16S rRNA sequencing and metabolomic analysis

Genomic DNA was extracted from rat fecal samples using the Magnetic Soil and Stool DNA Kit (Tiangen Biotech) and quantified via Qubit. The V3–V4 hypervariable region of the bacterial 16S rRNA gene was amplified using primers 341F/805R, purified with AMPure XP beads, and paired-end sequenced on a DNBSEQ-G99 platform (LC-Bio Technology).

For the metabolomic analysis of short-chain fatty acids (SCFAs), samples were extracted with pre-cooled 80% methanol and derivatized using EDC and 3-NPH. The supernatant was transferred into injection vials for LC-MS/MS analysis. Target analytes were separated on an Agilent Poroshell 120 EC-C18 column using an AB Sciex Jasper UPLC system coupled to a 4500MD triple quadrupole mass spectrometer operating in negative electrospray ionization (ESI−) mode. Raw metabolomic data were normalized and log-transformed prior to Principal Component Analysis (PCA) and Pearson correlation analysis to identify functional metabolic associations.

### 2.11 Statistical analysis

Statistical analyses were performed using GraphPad Prism software (version 10.6.0). Continuous data are presented as mean ± standard deviation (SD). The normality of data distribution and homogeneity of variances were assessed using the Shapiro-Wilk test and Levene’s test, respectively. For comparisons between two groups, the unpaired Student’s t-test was used for normally distributed data with equal variances, whereas Welch’s t-test was applied when variances were unequal. Non-normally distributed data were analyzed using the Mann-Whitney U test. For multiple-group comparisons, one-way ANOVA followed by Tukey’s *post-hoc* test was performed when variances were homogeneous. In cases of heterogeneous variances, Welch’s ANOVA followed by the Games-Howell *post-hoc* test was employed. Longitudinal data were analyzed using repeated-measures ANOVA. Correlations were assessed using Pearson’s correlation coefficient for normally distributed data and Spearman’s rank correlation coefficient for non-normally distributed data. A two-sided *P*-value < 0.05 was considered statistically significant.

## 3 Results

### 3.1 Longitudinal analysis of body weight

**Figure 3:**
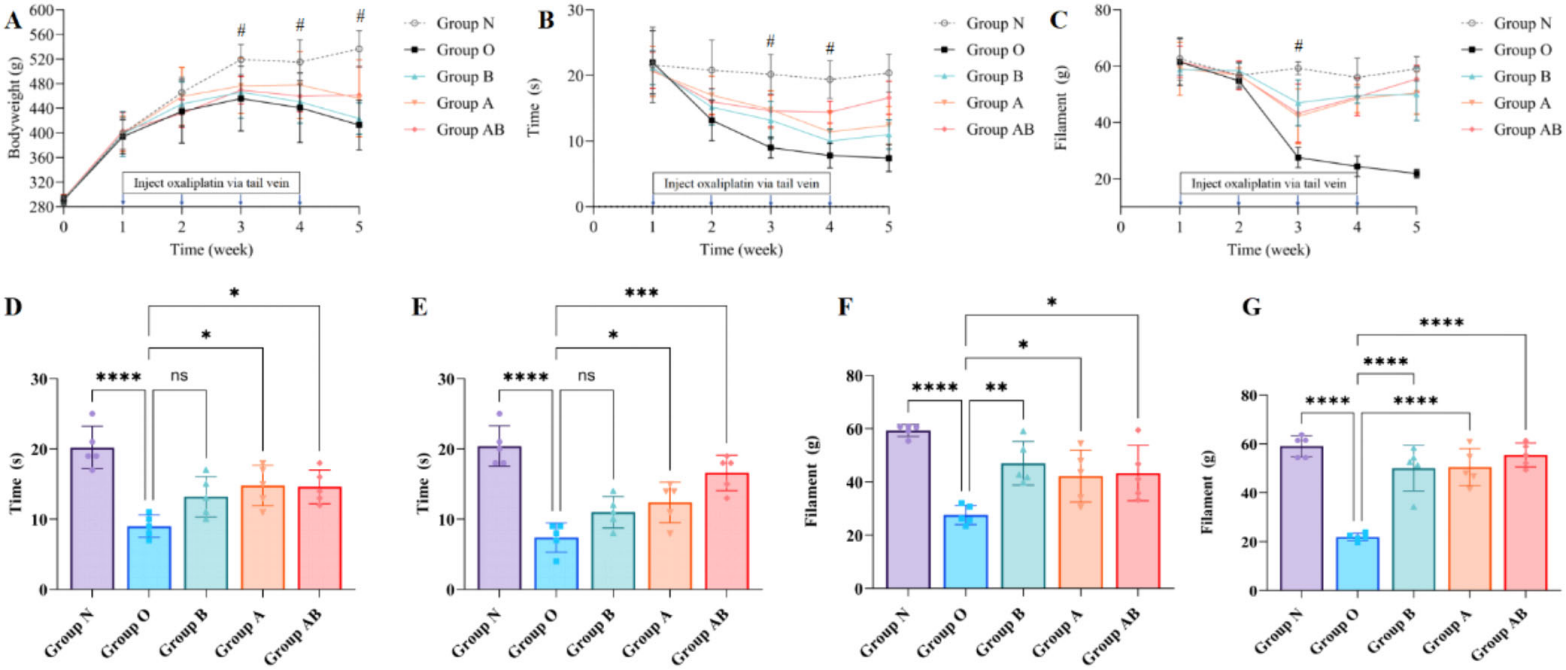
**(A)** The effect of oxaliplatin on body weight in different groups of rats. **(B)** Time course of cold allodynia development and treatment response. PWL in response to a 4 °C cold plate was measured over 5 weeks. **(C)** Temporal dynamics of mechanical nociception across experimental groups. **(D**, **E)** Therapeutic interventions with Group A and Group AB significantly alleviated cold hypersensitivity at Weeks 3 and 5, whereas sodium butyrate monotherapy (Group B) showed no significant improvement. **(F)** PWTs at week 3. Group O demonstrated significant hypersensitivity compared to Group (N) Therapeutic interventions significantly increased thresholds compared to Group O: Group B (** *P* = 0.0052); Group A (*P = 0.0453); Group AB (* *P* = 0.0285). **(G)** PWTs at week 5. All treatment groups demonstrated profound recovery compared to Group (O) No statistically significant differences were observed among treatment groups (A, B, AB) or between Group AB and Group N, indicating complete sensory restoration. Note: *P < 0.05, **P < 0.01, ****P < 0.0001; ns, not significant. #*P* < 0.05, indicating a statistically significant difference between Group AB and Group (N) Comparisons between two groups were analyzed using unpaired Student’s t-test. Statistical significance was determined by two-way repeated-measures ANOVA followed by *post-hoc* tests. N, Normal control; O, Oxaliplatin model; A, Oxaliplatin + *A. muciniphila*; B, Oxaliplatin + Sodium Butyrate; AB, Oxaliplatin + *A. muciniphila* + Sodium Butyrate.

Body weight was comparable across all groups at baseline. Beginning in week 3, the OIPN model group (Group O) exhibited a progressive decline in body weight relative to the normal control group (Group N). This difference increased over time, reaching statistical significance at week 3 (*P* = 0.05) and week 4 (*P* = 0.04), with the most substantial reduction observed at week 5 (*P* = 0.0007). At the week 5 endpoint, body weights in all treatment groups (Groups A, B, and AB) remained lower than in Group N (536.6 ± 29.7 g). However, the combined intervention (Group AB) showed the least severe body weight reduction among the treatment groups. Specifically, Group AB recorded the highest mean body weight (461.4 ± 46.7 g) with a comparatively lower level of statistical significance versus Group N (*P* = 0.0397) than Group A (456.2 ± 62.7 g, *P* = 0.0265) and Group B (423.4 ± 25.5 g, *P* = 0.0018). These results indicate that while the OIPN model induced a progressive and significant decline in body weight that was not fully restored to baseline control levels by any intervention, the combined therapy was the most effective at mitigating this loss (**Figure 3A**).

### 3.2 Cold allodynia

Cold hypersensitivity was assessed by measuring the PWL on a 4°C cold plate. A two-way repeated-measures ANOVA revealed significant main effects of time (F (4, 100) = 31.82, *P* < 0.0001) and intervention (F(4, 100) = 27.46, *P* < 0.0001). The oxaliplatin model group (Group O) exhibited a marked and sustained decrease in PWL compared to Group N, confirming successful induction of cold allodynia (**Figure 3B**). At week 3, Group O demonstrated significant cold hypersensitivity compared to Group N (*P* < 0.0001; **Figure 3D**). Regarding therapeutic interventions, Group A (*P* = 0.0163) and Group AB (*P* = 0.0212) significantly increased PWL thresholds compared to Group O, whereas monotherapy with sodium butyrate did not yield a statistically significant improvement (*P* = 0.1188). At week 5, Group O demonstrated significant cold hypersensitivity compared to Group N (*P* < 0.0001; **Figure 3E**). Regarding therapeutic interventions, Group A (*P* = 0.0388) and Group AB (*P* < 0.0001) significantly increased PWL thresholds compared to Group O, whereas monotherapy with sodium butyrate did not yield a statistically significant improvement (*P* = 0.2048).

### 3.3 Mechanical hypersensitivity in the OIPN model and response to therapy

Behavioral assessment of PWT confirmed the successful induction of OIPN. Rats in the model group (Group O) developed progressive mechanical hypersensitivity, reflected in a sustained decline in PWT. A two-way repeated-measures ANOVA revealed a significant interaction between time and group (F(16, 100) = 6.842, *P* < 0.0001), indicating divergent temporal trajectories of nociceptive sensitivity among groups (**Figure 3C**). Simple effects analysis revealed no significant differences among groups during weeks 1–2. From week 3 onwards, Group O exhibited a continuous and significant decline in PWTs compared to Group N. Groups A, B, and AB showed transient reductions at week 3, followed by a rebound to baseline levels during weeks 4–5. Group AB exhibited a “V-shaped” recovery pattern: despite a marked reduction at week 3, thresholds showed partial recovery at week 4 and near-normal levels were restored by week 5.

Therapeutic interventions (Groups A, B, and AB) significantly attenuated the development of mechanical allodynia compared to Group O. At week 3, significant intergroup differences were observed (one-way ANOVA: F = 11.18, *P* = 0.0004); all treatment groups had higher PWTs than Group O, though values remained below those of the normal control group (Group N) (**Figure 3F**). By week 5, this pattern shifted: all treatments conferred robust protection, with PWTs not significantly different from Group N (all *P* > 0.05) but markedly higher than Group O (all *P* < 0.0001) (**Figure 3G**).

The combined intervention (Group AB) demonstrated the most pronounced effect at this endpoint. Compared to Group O, Group AB maintained a significantly higher mean PWT (55.5 ± 4.9 g vs. 21.9 ± 1.5g, Welch’s t-test: t = 14.72, *P* < 0.0001), with a mean difference of 33.6 g (95% CI: 27.6 to 39.5). The exceedingly large effect size (η² = 0.978) indicates that the mechanical hypersensitivity in Group AB was effectively reversed to a level comparable with normal control (**Figure 3G**).

### 3.4 Systemic inflammatory profile and axonal damage marker

**Figure 4:**
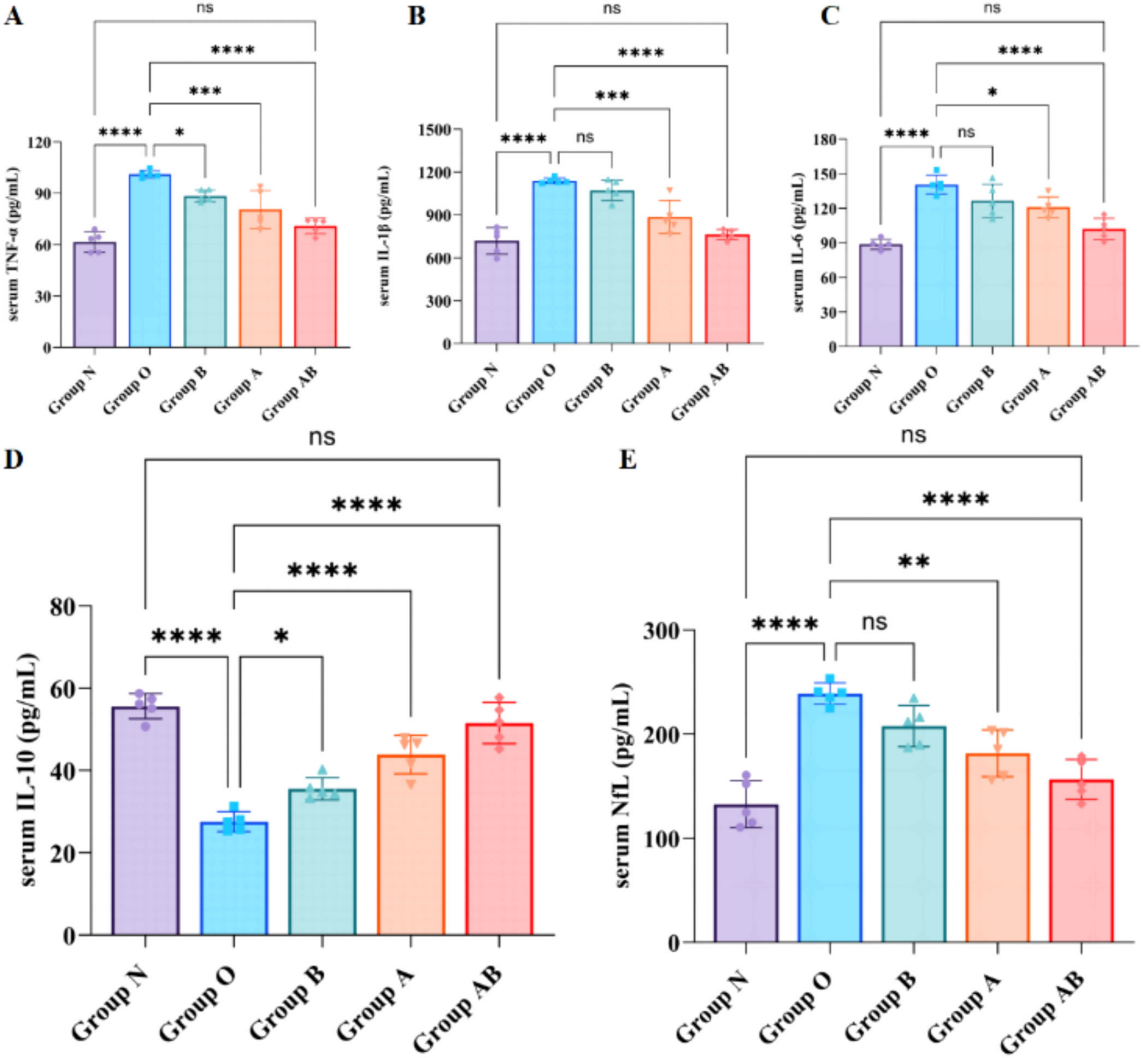
Reversal of oxaliplatin-induced systemic cytokine dysregulation. **(A-D)** Serum concentrations of pro-inflammatory cytokines A TNF-α, B IL-1β, C IL-6, and anti-inflammatory cytokine D IL-10. **(E)** Serum concentration of NfL. Serum samples were collected at the end of the experiment (Day 35, 24h after the final treatment). N, Normal control; O, Oxaliplatin model; A, Oxaliplatin + *A. muciniphila*; B, Oxaliplatin + Sodium Butyrate; AB, Oxaliplatin + *A. muciniphila* + Sodium Butyrate. Data are presented as mean ± SD (n = 5 per group). Statistical significance was determined by one-way ANOVA followed by Tukey’s *post hoc* test. *P < 0.05, **P < 0.01, ***P < 0.001, ****P < 0.0001, ns, not significant.

Oxaliplatin administration triggered a severe systemic inflammatory imbalance. Compared to Group N, the model group (Group O) exhibited significant elevations in pro-inflammatory cytokines (TNF-α, IL-1β, IL-6) and a reduction in the anti-inflammatory cytokine IL-10 (all *P* < 0.0001), indicating a shift toward a pro-inflammatory state (**Figures 4A–D**).

Therapeutic interventions differentially modulated this response. Group B significantly reduced TNF-α and increased IL-10, but did not significantly affect IL-1β or IL-6. Group A significantly lowered IL-1β and IL-6 while elevating IL-10. The combined intervention (Group AB) demonstrated the broadest efficacy, significantly suppressing all three pro-inflammatory cytokines and restoring IL-10 to a level comparable with Group N (*P* > 0.05).

Concurrently, serum NfL, a biomarker of axonal injury, was markedly elevated in Group O compared to Group N (*P* < 0.0001; **Figure 4E**). All treatment groups showed lower NfL levels than Group O. Among them, Group AB achieved the greatest reduction, resulting in NfL levels significantly lower than those in Group B (*P* < 0.05) and comparable to those in Group A (*P* > 0.05). The Group AB reduced NfL concentrations by 82.58 pg/mL (95% CI [58.84, 106.1]; *P* < 0.0001) with a substantial effect size (η*²* = 0.921). Normalization analysis indicated that Group AB restored NfL levels to 77.63% of the physiological baseline, a recovery rate significantly superior to that of Group A (53.71%) and Group B (29.37%). These data demonstrate that the combined regimen of *A. muciniphila* and sodium butyrate exerts superior neuroprotective effects, minimizing axonal injury more effectively than monotherapies.

### 3.5 Correlation analyses and comparative antineurodegenerative efficacy

**Figure 5:**
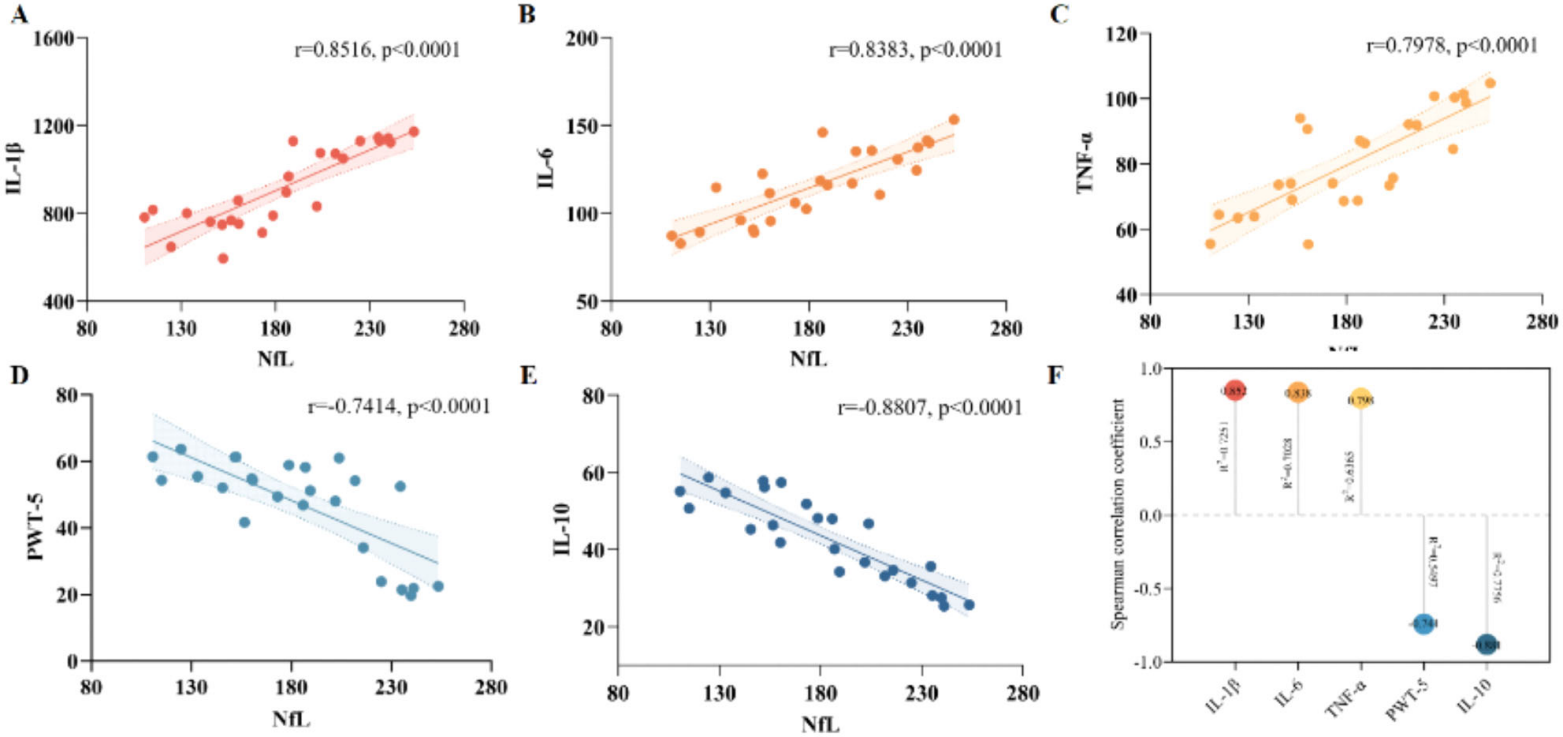
Linear regression assessed associations between serum NfL levels and key pathological indices. **(A–C)** Strong positive correlations between NfL and pro-inflammatory cytokines. **(D)** Inverse correlations between NfL and PWT at week 5. **(E)** Strong inverse correlation between NfL and the anti-inflammatory cytokine IL-10 (*r* = −0.881, *R²* = 0.776, *P* < 0.0001). **(F)** Summary visualization (Lollipop chart) ranking the strength of Pearson correlation coefficients (*r*) and coefficients of determination (*R²*). Elevated NfL levels closely mark the severity of systemic inflammation and functional sensory impairment. Shaded areas in scatter plots represent 95% confidence intervals of the regression lines.

Correlation analyses further elucidated the biological significance of elevated serum NfL levels (**Figure 5**). Robust positive correlations were observed between NfL and pro-inflammatory cytokines, with the strongest association identified for IL-1β (*r* = 0.852, *P* < 0.0001), followed by IL-6 and TNF-α (*r* > 0.79 for both). Conversely, NfL levels displayed a strong inverse correlation with the anti-inflammatory cytokine IL-10 (*r* = −0.881, *P* < 0.0001), accounting for 77.6% of the variance.

### 3.6 Histopathological assessment

**Figure 6:**
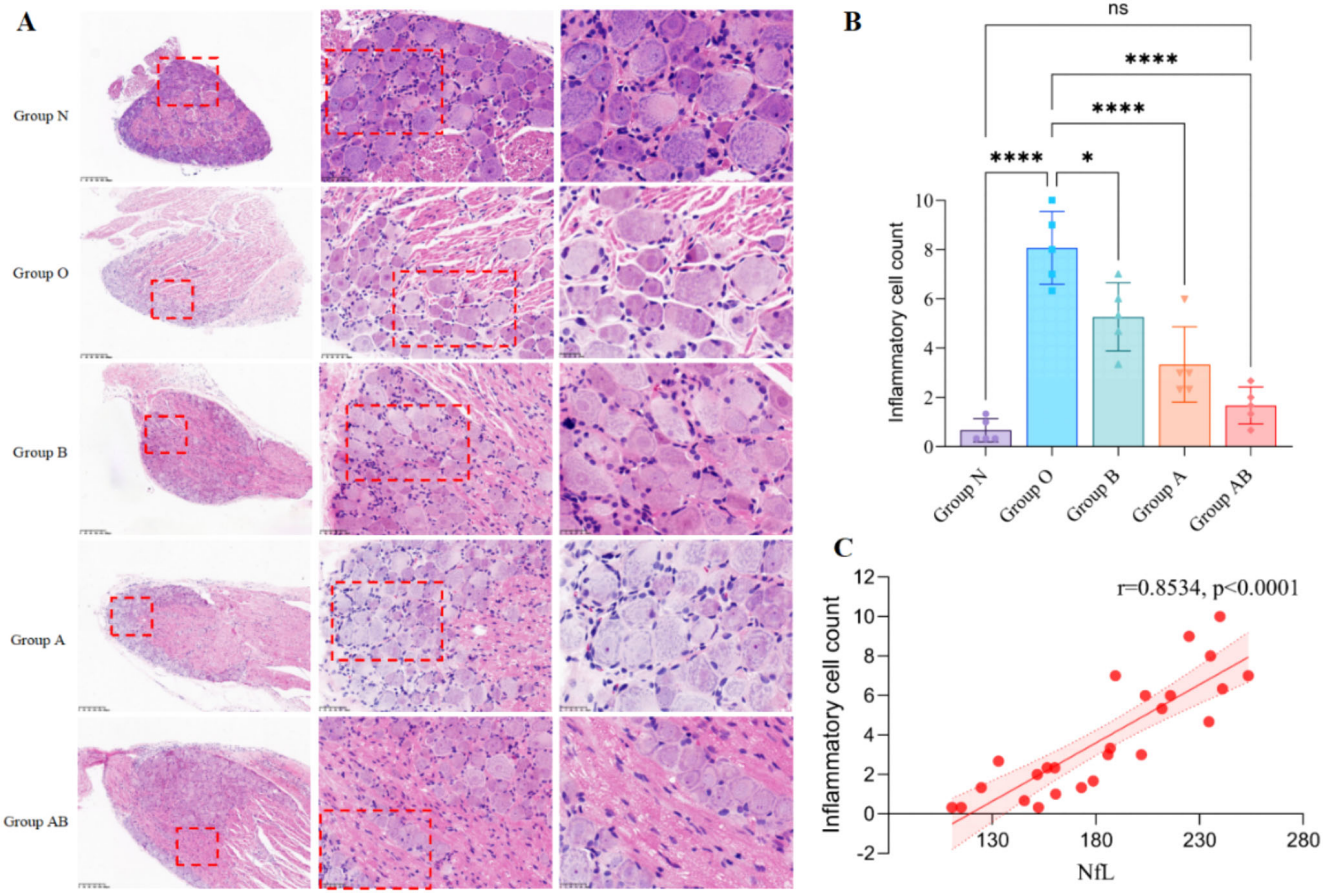
Histopathological evaluation of DRG and peripheral nerves. **(A)** Representative H&E-stained sections from L4–L5 DRG and sciatic nerve (or specified peripheral nerve) regions. Group N displays normal cytoarchitecture. **(B)** Bar chart comparing the number of inflammatory cells (lymphocytes, macrophages, and neutrophils) in rat DRG samples (400× magnification, Scale bar = 50μm). The inflammatory cell count was negligible in Group N. Group O exhibited a significant increase in inflammatory cell infiltration compared to Group N. All treatment groups (B, A, and AB) showed decreased inflammatory cell counts relative to Group O. Notably, the combined intervention group (Group AB) demonstrated the most substantial reduction, bringing the inflammatory cell count down to a level significantly lower than that of Group O and closest to the baseline levels observed in Group N. **(C)** Linear regression was used to assess the association between serum NfL levels and inflammatory cell counts. A strong positive correlation (r = 0.8534, *P* < 0.0001) was observed between NfL levels and inflammatory cells. *P < 0.05,****P < 0.0001; ns, not significant. Shaded areas in the scatter plots represent the 95% confidence intervals of the regression lines. Statistical significance was determined by two-way repeated-measures ANOVA followed by *post-hoc* tests. N, Normal control; O, Oxaliplatin model; A, Oxaliplatin + *A. muciniphila*; B, Oxaliplatin + Sodium Butyrate; AB, Oxaliplatin + *A. muciniphila* + Sodium Butyrate.

Histological analysis of L4–L5 DRG and peripheral nerves confirmed profound oxaliplatin-induced structural damage. Compared to Group N, which displayed normal neuronal and axonal architecture with intact myelin sheaths, the model group (Group O) exhibited severe degenerative pathology (**Figure 6**). This included neuronal atrophy with nuclear pyknosis, disorganization of satellite glial cells, axonal swelling and fragmentation, extensive myelin sheath disruption (delamination and demyelination), and marked interstitial edema with inflammatory cell infiltration. To enhance clarity, we have provided high-resolution images featuring specific arrow annotations in **Supplementary Figure 1**. These enlarged views allow for a more detailed examination of tissue morphology and cellular density.

All therapeutic interventions (Groups A, B, and AB) attenuated these morphological alterations. Treatment groups showed preserved neuronal architecture with reduced nuclear pyknosis, more compact axonal fibers with diminished swelling, and largely restored myelin integrity. Interstitial edema and inflammatory infiltration were also markedly reduced compared to Group O. Oxaliplatin administration induced a marked elevation in inflammatory cell infiltration relative to the normal control (Group O: 8.1 ± 1.5 vs. N: 0.7 ± 0.5, *P* < 0.0001; **Figure 6B**). While monotherapy with sodium butyrate (Group B: 5.3 ± 1.4) significantly attenuated inflammatory cell counts compared to the Group O (*P* = 0.0114), intervention with *A. muciniphila* alone (Group A: 3.3 ± 1.5) exerted a more potent inhibitory effect (*P* < 0.0001). Notably, the combined intervention (Group AB: 1.7 ± 0.7) demonstrated the most robust efficacy (*P* < 0.0001 vs. Group O), successfully restoring the inflammatory profile to levels statistically distinguishable from the normal control group (Group AB vs. N, *P* = 0.6819).

### 3.7 Quantification of intraepidermal nerve fiber density

**Figure 7:**
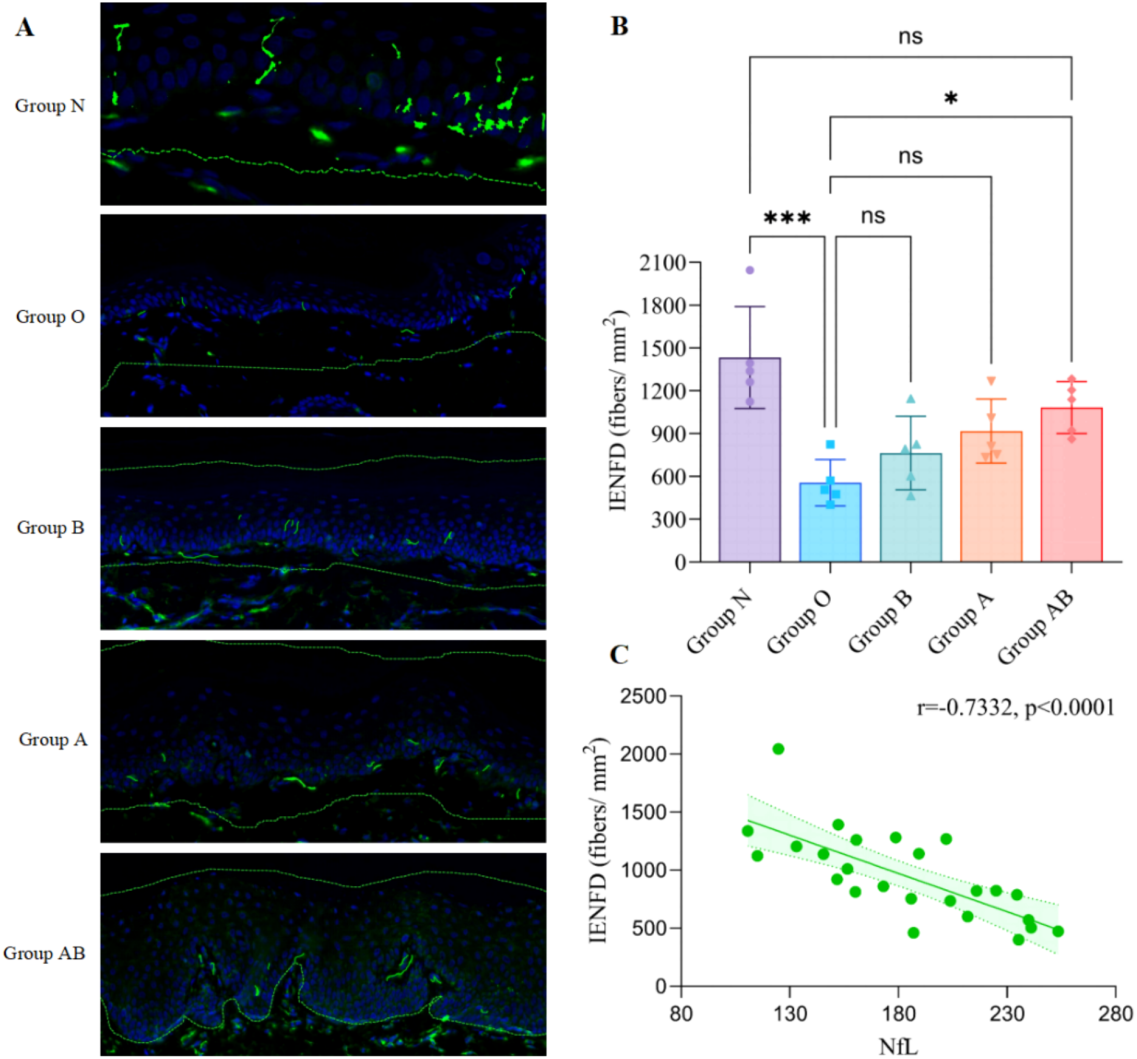
Immunofluorescence imaging and quantification of IENFD in rat hind paw plantar skin. **(A)** Representative images showing nerve fibers crossing the basement membrane into the epidermis (400× magnification, Scale bar = 25 μm). **(B)** Bar chart comparing IENFD levels among the different groups. Oxaliplatin administration precipitated a profound reduction in IENFD in Group O compared to the normal control (Group O vs. N, *P* < 0.0001). While monotherapies with *A. muciniphila* (Group A: P = 0.1785) and sodium butyrate (Group B: *P* = 0.6750) exhibited only marginal trends toward fiber preservation compared to the Model group, the combined intervention (Group AB) demonstrated significant neuroprotective efficacy (*P* = 0.0221 vs. Group O). Furthermore, the lack of statistical difference between Group AB and the normal control (Group AB vs. N, *P* = 0.2026) indicates that the combined therapeutic regimen effectively restored nerve terminal morphology. **(C)** Pearson correlation analysis revealing a significant negative correlation between IENFD and serum NfL levels (r = - 0.7332, *P* < 0.0001), indicating that lower nerve fiber density is associated with higher serum NfL. Density was calculated as the number of nerve fibers crossing the basement membrane divided by the total area of the tissue section analyzed. Data are presented as mean ± SD fibers/mm^2^. *P < 0.05, ***P < 0.0001; ns, not significant. N, Normal control; O, Oxaliplatin model; A, Oxaliplatin + *A. muciniphila*; B, Oxaliplatin + Sodium Butyrate; AB, Oxaliplatin + *A. muciniphila* + Sodium Butyrate.

To assess oxaliplatin-induced small-fiber neuropathy, we quantified IENFD in the hind paw plantar skin. Immunofluorescence imaging revealed a marked reduction of PGP9.5-positive nerve fibers in the model group (Group O) compared to normal control (Group N) (**Figure 7**).

Quantification confirmed a significant reduction in IENFD in Group O (555.2 ± 162.0 vs. 1432.0 ± 356.5 fibers/mm^2^ in Group N). Therapeutic interventions significantly attenuated this denervation. All treatment groups exhibited higher IENFD compared to Group O. The combined intervention Group AB and Group A showed comparable and superior protective efficacy (1082.0 ± 182.2 and 917.2 ± 224.6 fibers/mm^2^, respectively), both significantly exceeding the effect of Group B (762.8 ± 257.6 fibers/mm^2^). However, IENFD in all treatment groups remained lower than in Group N, indicating partial preservation.

### 3.8 Verification of *A. muciniphila* colonization and modulation of fecal SCFA

**Figure 8:**
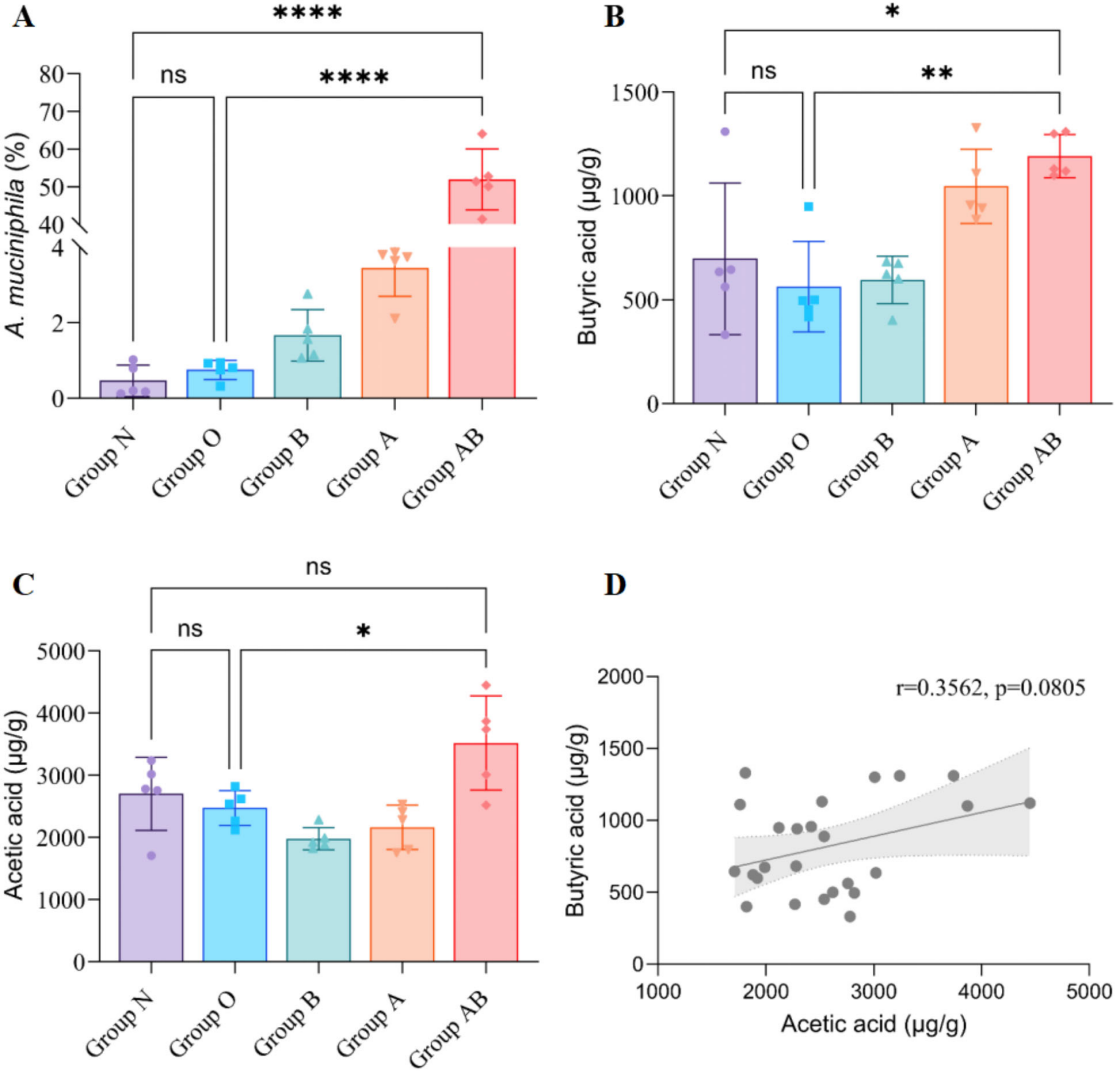
Verification of *A. muciniphila* colonization and modulation of fecal SCFA profiles. **(A)** Verification of *A. muciniphila* colonization. Relative abundance of the genus *Akkermansia* in fecal samples determined by 16S rRNA gene sequencing. **(B)** Quantification of fecal Butyrate content. Fecal Butyrate concentration was remarkably restored and elevated in the Combined Intervention Group AB, indicating the restoration of functional neuroprotective metabolites in the gut. **(C)** Quantification of fecal Acetate content. **(D)** Correlation analysis between fecal Acetate and Butyrate. Note: Data are presented as mean ± SD fibers/mm^2^. *P < 0.05, **P < 0.005, ****P < 0.0001; ns, not significant. N, Normal control; O, Oxaliplatin model; A, Oxaliplatin + *A. muciniphila*; B, Oxaliplatin + Sodium Butyrate; AB, Oxaliplatin + *A. muciniphila* + Sodium Butyrate.

To verify the efficacy of the gavage protocol, we performed 16S rRNA gene sequencing on fecal samples. As shown in **Figure 8**, the relative abundance of *A. muciniphila* was negligible (<1%) in both the Normal (N) and Model (O) groups. While the group receiving *A. muciniphila* alone (Group A) showed a modest increase in abundance (~3.5%) and the group treated with Sodium Butyrate alone (Group B) exhibited a slight elevation(~1.7%), the Combined Intervention group (Group AB) displayed a dramatic and statistically significant expansion of *A. muciniphila*, enabling it to become the dominant genus in the gut microbiome, reaching approximately 52% of total bacteria (*P* < 0.0001 compared to all other groups, **Figure 8A**). This confirms that the combined intervention successfully established high-abundance colonization.

Analysis of fecal SCFAs revealed that while the Model group showed no significant alteration in butyrate levels compared to the Normal group, the Combined intervention significantly elevated butyrate concentration (*P* < 0.005, **Figure 8B**), suggesting a functional boost of the neuroprotective microbial ecosystem. The metabolic profile of the Group AB—characterized by simultaneously elevated levels of both Acetate (a primary fermentation product of *A. muciniphila*) and Butyrate—supports the existence of a synergistic cross-feeding network. Quantification of fecal Acetate content (**Figure 8C**). The substantial production of acetate by the dominant *A. muciniphila* likely served as an abundant substrate for downstream butyrate-producing bacteria, thereby facilitating the overall restoration of SCFA levels. Furthermore, a correlation analysis indicated a positive trend between fecal acetate and butyrate levels (r = 0.3562), although this did not reach statistical significance (*P* = 0.0805, **Figure 8D**).

## 4 Discussion

OIPN remains a critical dose-limiting side effect in diverse chemotherapy regimens, with currently limited therapeutic options (6). In the present study, we demonstrate that the proposed Combined Intervention exerts a dual protective effect, simultaneously attenuating systemic toxicity—manifested as the suppression of inflammatory cytokines and mitigation of body weight loss—and alleviating neuropathic hypersensitivity. Crucially, these functional improvements were not transient but were closely underpinned by the structural preservation of IENFD. Collectively, our findings provide compelling evidence that this multi-modal strategy effectively halts the progression of OIPN, offering a potential therapeutic avenue to bridge the gap between neuroprotection and systemic recovery.

Crucially, this study confirms that the observed behavioral improvements (pain relief) are underpinned by genuine structural preservation of nerves, rather than symptomatic masking alone. To objectively quantify this axonal protection, we utilized serum NfL as a pharmacodynamic readout indicating structural integrity. Specifically, oxaliplatin administration triggered a marked elevation in serum NfL levels (*P* < 0.0001), which was significantly reversed by the combined intervention of *A. muciniphila* and sodium butyrate. This intervention reduced serum NfL levels by 34.53% compared to the model group, restoring them to 77.63% of the physiological baseline observed in healthy controls. In our analysis, the attenuation of serum NfL levels closely paralleled both behavioral recovery and the preservation of IENFD, validating NfL as a reliable indicator of both axonal damage intensity and therapeutic efficacy. This advances OIPN research in two critical dimensions. First, we elevate the clinical utility of NfL from a static “disease state marker” to a “pharmacodynamic biomarker,” facilitating the real-time monitoring of therapeutic responses—addressing a major limitation of prior studies that lacked post-treatment tracking (32). Second, we demonstrate that the combined modulation of gut microbiota and metabolites effectively reverses neuroinflammation and NfL elevation. This represents a distinct advantage over conventional single-agent strategies (e.g., neurotrophic agents or antioxidants), which have largely failed to achieve clinical translation (33, 34). While it is established that *A. muciniphila* and short-chain fatty acids exert immunomodulatory effects via the gut-brain axis (35, 36), the precise mechanistic synergies of their combination warrant further elaboration.

Mechanistically, we propose that the intervention counteracts the specific pathological cascade of OIPN. regarding the pathological inducer, Oxaliplatin exerts its neurotoxic effects primarily by accumulating in the DRG. Because the DRG lacks a protective blood-nerve barrier, platinum compounds readily accumulate in sensory neurons. This accumulation causes DNA platination, mitochondrial dysfunction, and oxidative stress, which ultimately trigger neuronal apoptosis and neuropathic pain (9–11). Beyond direct neurotoxicity, oxaliplatin compromises the intestinal barrier, facilitating the translocation of bacterial products—such as LPS—into blood circulation. This systemic endotoxemia activates TLR4/NF-κB signaling pathways, provoking a secondary wave of neuroinflammation in the spinal cord that exacerbates symptoms (37). Against this backdrop, *A. muciniphila* functions as the upstream modulator. Consistent with its documented role in regulating mucus metabolism (22, 23), our data confirmed that *A. muciniphila* treatment successfully rescued gut barrier integrity and reduced systemic LPS leakage. By acting as a “gatekeeper,” *A. muciniphila* effectively cuts off the supply of pro-inflammatory mediators. Importantly, *A. muciniphila* enrichment led to increased intestinal butyrate levels (38). Complementing this, NaB serves as the downstream direct effector. As the primary functional metabolite, NAB enters the circulation to target the nervous system. As a histone deacetylase inhibitor, NaB acts to suppress glial activation (microglia/astrocytes) and modulate pain pathways via PPAR-α or opioid signaling (25–27, 29). Notably, recent evidence also suggests that butyrate can mitigate chemotherapeutic side effects while potentially enhancing antitumor efficacy (39). Thus, the distinct actions of *A. muciniphila* and NaB constitute a cohesive “gut–metabolite–nerve” axis.

Synthesizing the observed interplay between serum NfL and systemic inflammatory mediators, alongside our histological findings, we propose a putative ‘Gut-Immune-Nerve’ cascade supported by recent literature. Initially, OIPN-associated dysbiosis likely compromises intestinal barrier integrity, facilitating a systemic inflammatory response as evidenced by the elevated serum cytokines (TNF-α, IL-1β, IL-6) in our model (40–42). This systemic surge promotes the infiltration of immune cells into the DRG—a process corroborated by our H&E density analysis—and ostensibly primes resident glial cells (43, 44). Although the present study focuses on the amelioration of neuroinflammation via gut microbiota and metabolites, previous studies indicate that such pro-inflammatory signals can activate local TLR4 signaling within the nerve, thereby establishing a positive feedback loop of cytokine release (26, 45). Consequently, this neurotoxic microenvironment acts as a primary driver for axonal degeneration, ultimately leading to the leakage of NfL into the circulation as a byproduct of cytoskeletal breakdown (14, 45, 46). Therefore, the significant reduction of serum NfL levels following the combined intervention likely reflects the successful interruption of this upstream inflammatory cascade, preserving neuro-structural integrity.

However, several limitations merit consideration. First, the restrictive sample size and inherent interspecies differences regarding chemotherapy regimens and genetic backgrounds necessitate caution in translational extrapolation. Future multi-center prospective studies are warranted to validate the clinical utility of serum NfL in OIPN cohorts, specifically by correlating it with long-term neurophysiological outcomes. Second, the current study lacked longitudinal monitoring across the differing phases of oxaliplatin administration. In future studies, we aim to optimize blood collection protocols—such as sampling prior to drug administration—to minimize animal stress and enable consistent longitudinal tracking. This will allow for the definition of NfL’s dynamic trajectory and the establishment of diagnostic cutoffs via ROC curve analysis, ultimately facilitating the early stratification of high-risk patients and guiding personalized neuropreventive strategies. Finally, we acknowledge the absence of a standard analgesic positive control group, such as gabapentin or duloxetine, in the current experimental design. While gabapentin is widely used for general neuropathic pain (47), current ASCO guidelines recommend duloxetine over gabapentin for CIPN due to the latter’s inconsistent efficacy data in chemotherapy-induced contexts (6). Furthermore, our study focused on the potential of the gut-brain axis intervention to exert neuroprotective and disease-modifying effects, which is mechanistically distinct from the symptomatic relief provided by ion channel blockers. Future investigations, which are currently underway in our laboratory, will include gabapentin or duloxetine as a comparator to evaluate the relative clinical efficacy of this microbial intervention.

## 5 Conclusion

In summary, this study demonstrates that oxaliplatin induces a spectrum of pathological alterations, including weight loss, pain hypersensitivity, structural nerve damage, and inflammatory dysregulation, which are concomitant with elevated serum NfL levels. Our findings indicate that serum NfL reflects both pain severity and inflammatory status, holding promise for the early diagnosis, severity assessment, and therapeutic monitoring of OIPN. Crucially, the combined administration of *A. muciniphila* and sodium butyrate significantly ameliorates mechanical and cold allodynia, suppresses systemic neuroinflammation, and attenuates axonal degeneration. This protective effect is substantiated by reduced serum NfL concentrations, thereby providing a novel strategy for treating OIPN by targeting the “gut-immune-nerve” axis.

## Data Availability

The raw data supporting the conclusions of this article will be made available by the authors, without undue reservation.

## References

[1] Bray F Laversanne M Sung H Ferlay J Siegel RL Soerjomataram I . Global cancer statistics 2022: GLOBOCAN estimates of incidence and mortality worldwide for 36 cancers in 185 countries. CA Cancer J Clin. (2024) 74:229–63. doi: 10.3322/caac.21834, PMID: 38572751

[2] McCabe MA Mauro AJ Schoen RE . Novel colorectal cancer screening methods - opportunities and challenges. Nat Rev Clin Oncol. (2025) 22:581–91. doi: 10.1038/s41571-025-01037-7, PMID: 40481325

[3] Hua Y Lv J Zhang Y Ding Y Chen J . LC-MS-based serum metabolomics analysis and potential biomarkers for oxaliplatin induced neurotoxicity in colorectal cancer. J Pharm BioMed Anal. (2024) 252:116492. doi: 10.1016/j.jpba.2024.116492, PMID: 39366306

[4] Gehr N Karlsson P Timm S Christensen S Hvid CA Peric J . Study protocol: fish oil supplement in prevention of oxaliplatin-induced peripheral neuropathy in adjuvant colorectal cancer patients - a randomized controlled trial. (OxaNeuro) BMC Cancer. (2024) 24:168. doi: 10.1186/s12885-024-11856-z, PMID: 38308227 PMC10837958

[5] Lemanska A Harkin A Iveson T Kelly C Saunders M Faithfull S . The association of clinical and patient factors with chemotherapy-induced peripheral neuropathy (CIPN) in colorectal cancer: secondary analysis of the SCOT trial. ESMO Open. (2024) 8:102063. doi: 10.1016/j.esmoop.2023.102063, PMID: 37988949 PMC10774973

[6] Loprinzi CL Lacchetti C Bleeker J Cavaletti G Chauhan C Hertz DL . Prevention and management of chemotherapy-induced peripheral neuropathy in survivors of adult cancers: ASCO guideline update. J Clin Oncol. (2020) 38:3325–48. doi: 10.1200/JCO.20.01399, PMID: 32663120

[7] Gehr NL Mortensen C Stage TB Pedersen MRV Rafaelsen SR Madsen JS . Neurofilament light chain as a marker for neuronal damage: integrating *in vitro* studies and clinical findings in patients with oxaliplatin-induced neuropathy. Cancer Chemother Pharmacol. (2025) 95:53. doi: 10.1007/s00280-025-04773-w, PMID: 40208334 PMC11985616

[8] Jia R Wan L Jin L Tian Q Chen Y Zhu X . Fucoidan reduces NET accumulation and alleviates chemotherapy-induced peripheral neuropathy via the gut-blood-DRG axis. J Neuroinflam. (2025) 22:100. doi: 10.1186/s12974-025-03431-5, PMID: 40186245 PMC11969723

[9] Desforges AD Hebert CM Spence AL Reid B Dhaibar HA Cruz-Topete D . Treatment and diagnosis of chemotherapy-induced peripheral neuropathy: An update. BioMed Pharmacother. (2022) 147:112671. doi: 10.1016/j.biopha.2022.112671, PMID: 35104697 PMC11118018

[10] Cesário FRAS de FranÃ§a JC Pereira AF Dias DBS de Oliveira AR Costa AS . Analgesic and neuroprotective effect of a lipid transfer protein isolated from Morinda citrifolia L. (noni) seeds on oxaliplatin-induced peripheral sensory neuropathy in mice. Naunyn Schmiedebergs Arch Pharmacol. (2025) 398:14515–35. doi: 10.1007/s00210-025-04216-6, PMID: 40304749

[11] Yang Y Zhao B Gao X Sun J Ye J Li J . Targeting strategies for oxaliplatin-induced peripheral neuropathy: clinical syndrome, molecular basis, and drug development. J Exp Clin Cancer Res. (2021) 40:331. doi: 10.1186/s13046-021-02141-z, PMID: 34686205 PMC8532307

[12] Misawa S Kogawa T Naito Y Suzuki T Takada M Denda T . A subgroup analysis of the MiroCIP study to evaluate chemotherapy-induced peripheral neuropathy: symptom profile, severity, and analgesia efficacy depending on type of chemotherapy. Expert Opin Pharmacother. (2025) 26:979–89. doi: 10.1080/14656566.2025.2499665, PMID: 40288415

[13] Andersen NE Boehmerle W Huehnchen P Stage TB . Neurofilament light chain as a biomarker of chemotherapy-induced peripheral neuropathy. Trends Pharmacol Sci. (2024) 45:872–9. doi: 10.1016/j.tips.2024.08.001, PMID: 39242335

[14] Le ND Muri L Grandgirard D Kuhle J Leppert D Leib SL . Evaluation of neurofilament light chain in the cerebrospinal fluid and blood as a biomarker for neuronal damage in experimental pneumococcal meningitis. J Neuroinflam. (2020) 17:293. doi: 10.1186/s12974-020-01966-3, PMID: 33028339 PMC7539528

[15] Khalil M Teunissen CE Lehmann S Otto M Piehl F Ziemssen T . Neurofilaments as biomarkers in neurological disorders - towards clinical application. Nat Rev Neurol. (2024) 20:269–87. doi: 10.1038/s41582-024-00955-x, PMID: 38609644

[16] Devarakonda SS Basha S Pithakumar A Thoshna LB Mukunda DC Rodrigues J . Molecular mechanisms of neurofilament alterations and its application in assessing neurodegenerative disorders. Ageing Res Rev. (2024) 102:102566. doi: 10.1016/j.arr.2024.102566, PMID: 39481763

[17] Kölliker Frers RA Otero-Losada M Kobiec T Udovin LD Aon Bertolino ML Herrera MI . Multidimensional overview of neurofilament light chain contribution to comprehensively understanding multiple sclerosis. Front Immunol. (2022) 13:912005. doi: 10.3389/fimmu.2022.912005, PMID: 35967312 PMC9368191

[18] Määttä LL Andersen ST Parkner T Hviid CVB Bjerg L Kural MA . Longitudinal change in serum neurofilament light chain in type 2 diabetes and early diabetic polyneuropathy: ADDITION-Denmark. Diabetes Care. (2024) 47:986–94. doi: 10.2337/dc23-2208, PMID: 38502878

[19] Ziemssen T Arnold DL Alvarez E Cross AH Willi R Li B . Prognostic value of serum neurofilament light chain for disease activity and worsening in patients with relapsing multiple sclerosis: results from the phase 3 ASCLEPIOS I and II trials. Front Immunol. (2022) 13:852563. doi: 10.3389/fimmu.2022.852563, PMID: 35432382 PMC9009385

[20] Xu J Lu L Jiang S Qin Z Huang J Huang M . Paeoniflorin ameliorates oxaliplatin-induced peripheral neuropathy via inhibiting neuroinflammation through influence on gut microbiota. Eur J Pharmacol. (2024) 971:176516. doi: 10.1016/j.ejphar.2024.176516, PMID: 38513881

[21] Chen X Gan Y Au NPB Ma CHE . Current understanding of the molecular mechanisms of chemotherapy-induced peripheral neuropathy. Front Mol Neurosci. (2024) 17:1345811. doi: 10.3389/fnmol.2024.1345811, PMID: 38660386 PMC11039947

[22] Cani PD Depommier C Derrien M Everard A de Vos WM . *Akkermansia muciniphila*: paradigm for next-generation beneficial microorganisms. Nat Rev Gastroenterol Hepatol. (2022) 19:625–37. doi: 10.1038/s41575-022-00631-9, PMID: 35641786

[23] Ioannou A Berkhout MD Geerlings SY Belzer C . *Akkermansia muciniphila*: biology, microbial ecology, host interactions and therapeutic potential. Nat Rev Microbiol. (2024) 23:162–77. doi: 10.1038/s41579-024-01106-1, PMID: 39406893

[24] Qiao CM Huang WY Zhou Y Quan W Niu GY Li T . *Akkermansia muciniphila* Is Beneficial to a Mouse Model of Parkinson’s Disease, via Alleviated Neuroinflammation and Promoted Neurogenesis, with Involvement of SCFAs. Brain Sci. (2024) 14:238. doi: 10.3390/brainsci14030238, PMID: 38539626 PMC10968773

[25] Xu K Wang G Gong J Yang X Cheng Y Li D . *Akkermansia muciniphila* protects against dopamine neurotoxicity by modulating butyrate to inhibit microglia-mediated neuroinflammation. Int Immunopharmacol. (2025) 152:114374. doi: 10.1016/j.intimp.2025.114374, PMID: 40056512

[26] Sun J Lu L Lian Y Xu S Zhu Y Wu Y . Sodium butyrate attenuates microglia-mediated neuroinflammation by modulating the TLR4/MyD88/NF-κB pathway and microbiome-gut-brain axis in cardiac arrest mice. Mol Brain. (2025) 18:13. doi: 10.1186/s13041-025-01179-w, PMID: 39962509 PMC11834616

[27] Guo TT Zhang Z Sun Y Zhu RY Wang FX Ma LJ . Neuroprotective effects of sodium butyrate by restoring gut microbiota and inhibiting TLR4 signaling in mice with MPTP-induced parkinson’s disease. Nutrients. (2023) 15:930. doi: 10.3390/nu15040930, PMID: 36839287 PMC9960062

[28] Ho IHT Zou Y Luo K Qin F Jiang Y Li Q . Sodium butyrate restored TRESK current controlling neuronal hyperexcitability in a mouse model of oxaliplatin-induced peripheral neuropathic pain. Neurotherapeutics. (2025) 22:e00481. doi: 10.1016/j.neurot.2024.e00481, PMID: 39542827 PMC11742850

[29] Avagliano C De Caro C Cuozzo M Roberti R Russo E La Rana G . Sodium Butyrate ameliorates pain and mood disorders in a mouse model of Parkinson disease. BioMed Pharmacother. (2025) 184:117903. doi: 10.1016/j.biopha.2025.117903, PMID: 39938349

[30] Höke A Ray M . Rodent models of chemotherapy-induced peripheral neuropathy. ILAR J. (2014) 54:273–81. doi: 10.1093/ilar/ilt053, PMID: 24615440

[31] Lauria G Hsieh ST Johansson O Kennedy WR Leger JM Mellgren SI . European Federation of Neurological Societies/Peripheral Nerve Society Guideline on the use of skin biopsy in the diagnosis of small fiber neuropathy. Report of a joint task force of the European Federation of Neurological Societies and the Peripheral Nerve Society. Eur J Neurol. (2010) 17:903–12. doi: 10.1111/j.1468-1331.2010.03023.x, PMID: 20642627

[32] Kim SH Choi MK Park NY Hyun JW Lee MY Kim HJ . Serum neurofilament light chain levels as a biomarker of neuroaxonal injury and severity of oxaliplatin-induced peripheral neuropathy. Sci Rep. (2020) 10:7995. doi: 10.1038/s41598-020-64511-5, PMID: 32409710 PMC7224372

[33] Kawashiri T Mine K Kobayashi D Inoue M Ushio S Uchida M . Therapeutic agents for oxaliplatin-induced peripheral neuropathy; experimental and clinical evidence. Int J Mol Sci. (2021) 22:1393. doi: 10.3390/ijms22031393, PMID: 33573316 PMC7866815

[34] Warren G Osborn M Tsantoulas C David-Pereira A Cohn D Duffy P . Discovery and preclinical evaluation of a novel inhibitor of FABP5, ART26.12, effective in oxaliplatin-induced peripheral neuropathy. J Pain. (2024) 25:104470. doi: 10.1016/j.jpain.2024.01.335, PMID: 38232863 PMC12993615

[35] Blacher E Bashiardes S Shapiro H Rothschild D Mor U Dori-Bachash M . Potential roles of gut microbiome and metabolites in modulating ALS in mice. Nature. (2019) 572:474–80. doi: 10.1038/s41586-019-1443-5, PMID: 31330533

[36] AlKady AR Abd Elmaaboud MA Eltantawy AF Abdin AA . The potential amelioration of oxaliplatin-induced peripheral neuropathy in Sprague Dawley rats by sodium butyrate via targeting neuro-immuno-inflammatory axis. Toxicol Appl Pharmacol. (2025) 506:117651. doi: 10.1016/j.taap.2025.117651, PMID: 41271030

[37] Zhang Z Ye J Liu X Zhao W Zhao B Gao X . Huangqi Guizhi Wuwu decoction alleviates oxaliplatin-induced peripheral neuropathy via the gut-peripheral nerve axis. Chin Med. (2023) 18:114. doi: 10.1186/s13020-023-00826-5, PMID: 37679804 PMC10485938

[38] Ramakrishna C Corleto J Ruegger PM Logan GD Peacock BB Mendonca S . Dominant role of the gut microbiota in chemotherapy induced neuropathic pain. Sci Rep. (2019) 9:20324. doi: 10.1038/s41598-019-56832-x, PMID: 31889131 PMC6937259

[39] Mukhopadhya I Louis P . Gut microbiota-derived short-chain fatty acids and their role in human health and disease. Nat Rev Microbiol. (2025) 23:635–51. doi: 10.1038/s41579-025-01183-w, PMID: 40360779

[40] Dehghani S Khorsandi H Hosseinzadegan R Rahimi H Mottaghi M Fallahpour S . Neuroinflammatory mechanisms and therapeutic targets in oxaliplatin-induced peripheral neuropathy: a comprehensive review. Neurotox Res. (2025) 43:52. doi: 10.1007/s12640-025-00775-x, PMID: 41389089

[41] Wei H Yu C Zhang C Ren Y Guo L Wang T . Butyrate ameliorates chronic alcoholic central nervous damage by suppressing microglia-mediated neuroinflammation and modulating the microbiome-gut-brain axis. BioMed Pharmacother. (2023) 160:114308. doi: 10.1016/j.biopha.2023.114308, PMID: 36709599

[42] Chen C Xu JL Gu ZC Zhou SS Wei GL Gu JL . Danggui Sini decoction alleviates oxaliplatin-induced peripheral neuropathy by regulating gut microbiota and potentially relieving neuroinflammation related metabolic disorder. Chin Med. (2024) 19:58. doi: 10.1186/s13020-024-00929-7, PMID: 38584284 PMC10999090

[43] Lund H Hunt MA Kurtović Z Sandor K Kägy PB Fereydouni N . CD163+ macrophages monitor enhanced permeability at the blood-dorsal root ganglion barrier. J Exp Med. (2024) 221:e20230675. doi: 10.1084/jem.20230675, PMID: 38117255 PMC10733632

[44] Lacagnina MJ Willcox KF Boukelmoune N Bavencoffe A Sankaranarayanan I Barratt DT . B cells drive neuropathic pain-related behaviors in mice through IgG-Fc gamma receptor signaling. Sci Transl Med. (2024) 16:eadj1277. doi: 10.1126/scitranslmed.adj1277, PMID: 39321269 PMC11479571

[45] Zhang P Yang M Chen C Liu L Wei X Zeng S . Toll-like receptor 4 (TLR4)/opioid receptor pathway crosstalk and impact on opioid analgesia, immune function, and gastrointestinal motility. Front Immunol. (2020) 11:1455. doi: 10.3389/fimmu.2020.01455, PMID: 32733481 PMC7360813

[46] Ripamonti E Gisslén M Hagberg L Bathala P Kale S Stengelin M . Cerebrospinal fluid biomarkers associated with neurofilament light levels: A study in HIV disease. J Neuroimmunol. (2025) 400:578521. doi: 10.1016/j.jneuroim.2025.578521, PMID: 39914269

[47] Mauermann ML Staff NP . Peripheral neuropathy: A review. JAMA. (2026) 335:255–66. doi: 10.1001/jama.2025.19400, PMID: 41247746

