# Supplementary material for: Combined intervention of Akkermansia muciniphila and sodium butyrate ameliorates oxaliplatin-induced peripheral neuropathy by suppressing neuroinflammation and reducing serum neurofilament light chain
Source: Front Immunol. 2026 Mar 4;17:1787012. doi: 10.3389/fimmu.2026.1787012 (PMC12995751; doi:10.3389/fimmu.2026.1787012)
Supplement: Supplementary file 1 [file Image1.pdf]

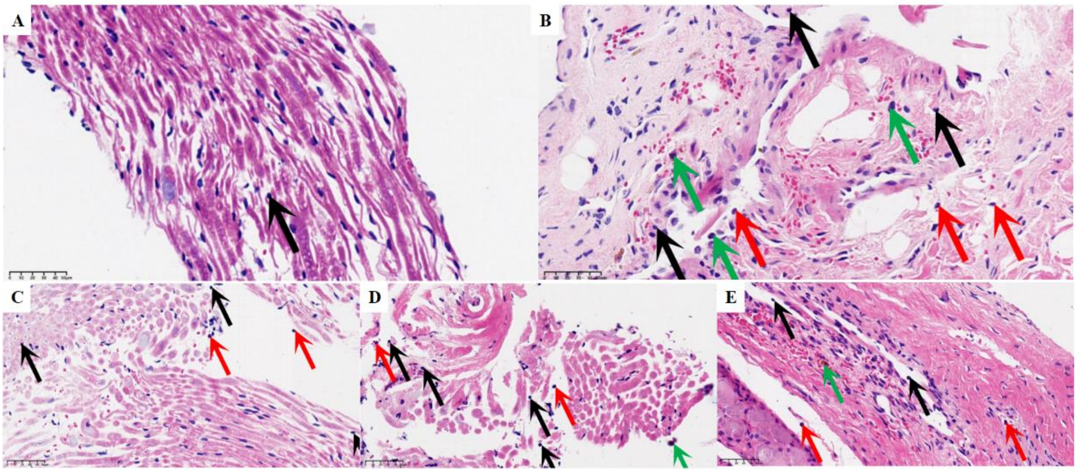

FIGURE S1

Representative images of inflammatory cells in each group. **A** Group N; **B** Group O; **C** Group A; **D** Group B; **E** Group AB. Black arrows: lymphocytes; red arrows: neutrophils; green arrows: macrophages.
